# Inactivation combined with cell lysis of Pseudomonas putida using a low pressure carbon dioxide microbubble technology

**DOI:** 10.1002/jctb.5299

**Published:** 2017-05-12

**Authors:** Ali R Mulakhudair, Mahmood Al‐Mashhadani, James Hanotu, William Zimmerman

**Affiliations:** ^1^ Department of Chemical and Biological Engineering University of Sheffield Sheffield UK; ^2^ The University of Babylon The Ministry of Higher Education and Scientific Research Iraq; ^3^ Department of Chemical Engineering University of Baghdad Baghdad Iraq

**Keywords:** inactivation, cell lysis, CO_2_, microbubbles, Pseudomonas

## Abstract

**BACKGROUND:**

Inactivation processes can be classified into non‐thermal inactivation methods such as ethylene oxide and γ‐radiation, and thermal methods such as autoclaving. The ability of carbon dioxide enriched microbubbles to inactivate Pseudomonas putida suspended in physiological saline, as a non‐thermal sterilisation method, was investigated in this study with many operational advantages over both traditional thermal and non‐thermal sterilisation methods.

**RESULTS:**

Introducing carbon dioxide enriched microbubbles can achieve ∼2‐Log reduction in the bacterial population after 90 min of treatment, addition of ethanol to the inactivation solution further enhanced the inactivation process to achieve 3, 2.5 and 3.5‐Log reduction for 2%, 5% and 10 %( v/v) ethanol, respectively. A range of morphological changes was observed on Pseudomonas cells after each treatment, and these changes extended from changing cell shape from rod shape to coccus shape to severe lesions and cell death. Pseudomonas putida KT 2440 was used as a model of gram‐negative bacteria.

**CONCLUSION:**

Using CO_2_ enriched microbubbles technology has many advantages such as efficient energy consumption (no heat source), avoidance of toxic and corrosive reagents, and in situ treatment. In addition, many findings from this study could apply to other gram‐negative bacteria. © 2017 The Authors. *Journal of Chemical Technology & Biotechnology* published by John Wiley & Sons Ltd on behalf of Society of Chemical Industry.

## INTRODUCTION

Traditionally, inactivation (sterilisation) methods can be classified into two main classes: thermal and non‐thermal sterilisation methods. Thermal methodologies, such as autoclaving and steam sterilisation, have been widely used to sterilise a variety of materials such as nutritional materials, medical devices and watery solutions. Other substances cannot be sterilised using these methodologies due to their heat‐sensitive compositions.^1^ Alternatively, non‐thermal methodologies have been applied on heat‐labile materials such as high protein foods as they can retain all their physical, mechanical and optical characteristics without denatured changes.^1^ Thermal methods such as steam sterilisation, however, have higher capital and operating costs than non‐thermal methods. On the other hand, thermal methods have slightly higher effectiveness than the non‐thermal methodologies.^2^ Therefore, there are increasing demands for non‐thermal sterilisation methods such as those using ethylene oxide, γ irradiation and supercritical CO_2_.^2^, ^3^, ^4^

However, these methods still have drawbacks, such as the toxicity and carcinogenicity of ethylene oxide,^2^ and changing the mechanical characteristics of biomaterials by γ‐irradiation.^3^ In contrast using carbon dioxide can avoid many of these drawbacks. Recently, CO_2_ has been used to preserve foodstuffs and to inactivate a wide range of microorganisms including yeasts and bacteria. Other potential applications for this methodology include *in situ* inactivation processes for both pretreatment slurry of lignocellulosic biomass after microbial pre‐treatment and contaminated algal culture. The primary action of using carbon dioxide as an effective inactivation technology is instigated by changing the balance of biological systems within the cells.^4^ The proposed targets for CO_2_ activity were reviewed and summarised previously.^5^

CO_2_ has a strong tendency to dissolve in water, which increases with hydrophobic solutions and at low temperatures. Interestingly, the CO_2_ solubility coefficient increases with decreasing temperature, and at 15 °C, the CO_2_ solubility coefficient is ∼1.^6^ The CO_2_ activity can be quantified using the survivor ratio, calculated using the following equation suggested previously:^7^

(1) $$\log\frac{N}{\text{No}}=k.t\;2.303$$

where *N* is the number of bacterial cells (colony forming units mL^−1^), *N_0_* is the initial number of bacterial cells (colony forming units mL^−1^), *k* is the sterilisation rate constant min ^−1^(*D*‐value) and *t* is the time (min).

The inactivation capability of CO_2_ depends on the dissolved CO_2_ concentration, while the pressure of pressurized CO_2_ might not have a direct effect on the microbial inactivation, it influences its dissolution of CO_2_.

*Pseudomonas putida* KT2440 is a gram‐negative, aerobic rod‐shaped bacterium, with a significant metabolic diversity. It is adapted to different environments such as soils, aquatic systems and rhizosphere.^8^ Its unique traits make it suitable as a bio‐control and growth‐promoting agent^9^ and therefore model organism for the current study.

The efficacy of this pre‐treatment process depends on the diffusion coefficient of carbon dioxide, which can be controlled by the contact time and interfacial area. The low rise velocity of microbubbles, coupled with their high surface area to volume ratio, provide superior mass transfer. To enhance mass transfer, CO_2_ needs to be introduced into the bulk liquid as microbubbles.^10^ In addition, microbubbles can generate free radicals during their collapse.^11^, ^12^ These radicals have a much higher standard redox potential (2.80 V) than oxidants such as ozone and hydrogen peroxide (2.07 and 1.77 V, respectively), and thus, they show instant and non‐selective reactivity with the majority of organic compounds present in solution.^13^, ^14^

pH influences not only the quantity of free radicals generated by the collapsed microbubbles but also the electrostatic profile of the microbubbles and the degree of ionization of organic compounds in aqueous solution.^12^ At pH above 4.5, the zeta (ζ) potential of microbubbles tends to have a negative sign, with the magnitude varying directly with increasing pH.^15^, ^16^ For values of pH below 4.5, the zeta potential has a positive value, and it increases with decreasing pH, resulting in a higher probability of negative charged compounds such as lipopolysaccharides (LPS) in the outer membrane of gram‐negative bacteria;^17^ which can approach the bubble interface and accelerate the reaction processes.^12^ For *P. putida*, the isoelectric point occurs at pH 3,^18^ and below this value, the net surface charge is positive, but negative otherwise.^19^ However, the pH dependent adhesion cannot be described as a linear function as shown previously.^19^ Based on *P. putida* average cell size: 0.5 to 0.6 µm in diameter and from 1.4 to 1.7 µm in length,^20^ it was calculated that one bacterium covers up to 1 µm^2^ of surface. However, the surface of each microbubble represents 7850 µm^2^, using 50 µm as the average microbubbles size. Thus, the adhesion intensity attained in the present experiments is sufficient to attach many bacterial cells, helping to direct diffusion of CO_2_ in aqueous phase via a bacterial membrane. CO_2_ can diffuse through the cell membrane of bacterial cells to accumulate in the phospholipid layer, leading to increased fluidity of plasma membrane and causing an anaesthesia effect for microbial cells.^21^

The aim of the present study is to explore the inactivation of *P. putida* using CO_2_ enriched microbubbles. Wild‐type cells were treated with CO_2_ microbubbles at low pressure and temperatures. Additives were used to enhance the activity of this process and to achieve a lower survivor ratio (greater log reduction [− Log]).

## MATERIAL AND METHODS

*Pseudomonas putida* was cultivated at 30 °C for 24 h on the nutrient broth (Sigma–Aldrich, UK). Normal saline solution (0.85%) was prepared and refrigerated for 24 h at 6 °C. It is worth mentioning that normal saline is the generally favoured fluid as it is isotonic and less likely to interfere with the inactivation process. Diluted bacterial culture was prepared by mixing 900 mL of sterile cold saline with 100 mL of bacterial culture, which was grown for 18–24 h, and the final temperature set at 6 °C.

**Figure 1:**
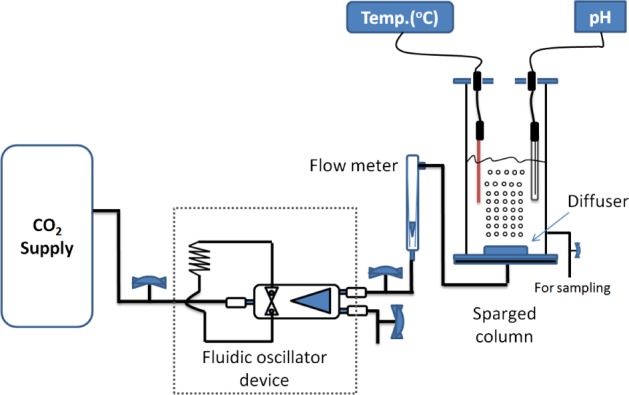
Schematic representation of the experimental set‐up. Pure CO_2_ gas (100%) is fed into the microbubbles diffuser.

The reactor was connected to a CO_2_ (100%) gas cylinder (BOC, UK) through a diffuser (ceramic diffuser, point four systems Inc. UK), and the reactor has a final capacity 1.5 L (Fig. 1). These experiment sets were conducted for 90 min (time required to reach equilibrium state according to the preliminary studies), and samples were drawn every 15 min except the first sample, which was drawn after 7 mins. pH and temperature profiles were measured at the same time as drawing samples. Drawn samples were diluted with sterile normal saline and aseptically streaked on nutrient agar plates using an inoculation loop and incubated at 30 °C for 24 h, and thereafter; the grown colonies were counted using two software packages: ColonyCount, 2015 © Promega Corporation and HGColonyLT, 2014 © HyperGEAR Inc. Petri dishes with 30–300 colonies were counted, and colony‐forming unit per mL was calculated for each sample. In addition, a colony counter (Bio Spectrum 410 imaging system, UVP, UK) was also used to verify and correct the colony count. Survivor ratio was calculated and plotted using ln (N/N_0_) on Y‐axis and time on X‐axis, where N refers to the number of colony forming units for treated samples, N_0_ refers to the initial number of colony forming units before CO_2_ treatment.

In addition, the highest microbubble population fraction generated was of size less than 100 microns and the addition of ethanol to the treatment solution did not affect the bubble size significantly, as both chemicals were added at low concentrations and this has a very limited effect on the size of microbubbles.

### Morphological examination of the bacterial cells

#### *Combined microscopy*

For each experiment, two slides for bacterial smear before and after the inactivation process were prepared and stained with gram stain according to the standard protocol described previously.^22^

#### *Scanning electron microscopy*

Microbial specimens were fixed chemically for examination with scanning electron microscopy (SEM). Initially, the specimens were kept in normal saline to avoid any changes as a result of osmolality as normal saline is isotonic solution. Thereafter, the specimens were mounted on 12.5 mm diameter stubs, attached with carbon‐sticky tabs, and then coated using an Edwards S150B sputter coater with approximately 25 nm of gold. The specimens were examined in a Philips XL‐20 scanning electron microscope at an accelerating voltage of 20 kV.

### **Determination of** CO_2_ **concentration**

Kinetically, conversion of CO_2_ into carbonic acid is very slow, and _∼_0.2% of CO_2_ can be converted to carbonic acid and its respective ions, while 99.8% of CO_2_ tends to remain as a dissolved gas, which can be shown in the dissociated equilibrium constraint:^23^

(2) $$CO_{2}\left(g\right)\;↔\;CO_{2}\left(aq\right)$$

(3) $$CO_{2}\left(aq\right)\;+H_{2}O\;↔\;H_{2}CO3$$

(4) $$K_{h}=\left[H_{2}CO_{3}\right]/\left[CO_{2\left(aq\right)}\right]$$

Carbonic acid is a diprotic acid, containing two hydrogen atoms ionizable in water and dissociates into bicarbonate and carbonate ions according to the following equations:

(5) $$H_{2}CO_{3}\;↔\;HC{O^{-}}_{3}+H^{+}$$

(6) $$HC{O^{-}}_{3}\;↔C{O^{-}}_{3}+H^{+}$$

Obviously, it is possible to infer the concentration of dissolved CO_2_ from the pH assuming equilibrium and a well‐mixed system.

The equilibrium constraint for each dissociation reaction (5) and (6) are:

(7) $$K_{a1}=\left[H{O^{-1}}_{3}\right]\left[H^{+}\right]/\left[H_{2}CO_{3}\right]$$

(8) $$K_{a2}=\left[C{O_{3}}^{-2}\right]\left[H^{+}\right]/\left[HC{O^{-}}_{3}\right]$$

The system should satisfy the electroneutrality constraint, therefore

(9) $$\left[H\right]=\left[OH^{-}\right]+\left[HC{O^{-}}_{3}\right]+2\left[C{O^{-2}}_{3}\right]$$

Dissociation of water to hydrogen and hydroxide ions with dissociation of CO_2_ can give five equations and the definitions of the pH relate with six unknowns, which are [H^+^], [OH^−^], [HCO_3_^−^], [H_2_CO_3_], [CO_3_^2−^] and [CO_2__aq_], which can be solved through a set of nonlinear algebraic equations (Table 1). Accordingly, CO_2_ concentration can be inferred from pH measurements as follows:

(10) $$\left[CO_{2\left(aq\right)\;}\right]=\frac{(10^{-pH})\left({\left(10^{-pH}\right)}^{2}-10^{-14}\right)}{K_{a1}K_{h\;}\left[10^{-pH}\right]+2K_{a1}K_{a2}K_{h}}$$

Therefore, the concentration of dissolved CO_2_ was measured using Equation (10) based on pH values measured by Mettler Toledo™ S220 (pH) meter. It is worth noting that addition of ethanol to the inactivation solution did not influence the pH of the solution and thus, the same Equation (10) was used to calculate the concentration of dissolved CO_2_ in the experimental sets with ethanol used as an additive.

**Table 1:** Chemical reactions with their corresponding reaction rate constants used to integrate the CO_2_ concentration equations in the current study

Chemical reaction	Reaction rate constant	Unit	Reference
$H_{2}O\;\overset{K^{f}}{\to }\;H^{+}\;+\;OH^{-}$	*K* ^*f*^ = 5.5 × 10^− 6^	1/*s*	24, 25, 26
$H^{+}\;+\;OH^{-\;}\overset{K^{r}}{\to }\;H_{2}O$	*K* ^*r*^ = 3 × 10^3^ *K*_*eq*_ = 1.8 × 10^− 16^ *K*_*w*_ = 1 × 10^− 14^	1/*mole*/*sec*	
*CO*_2_+ $H_{2}O\;\overset{K^{f}}{\to }\;H_{2}CO_{3}$	*K* ^*f*^ = 0.043	1/*mole*/*sec*	27
$H_{2}C0_{3\;}\overset{K^{r}}{\to }$ *CO*_2_ + *H*_2_ *O*	*K* ^*r*^ = 14.98	1/*s*	
	*K*_*eq*_ = 2.87 × 10^− 3^		
$H_{2}C0_{3\;}\overset{K^{r}}{\to }$ *HCO*_3_ + *H*	*K* ^*f*^ = 10^6.9^		
$HC0_{3}\;+\;H^{+}\overset{K^{r}}{\to }\;H_{2}C0_{3}$	*K* ^*r*^ = 4.67 × 10^10^	1/*mole*/*sec*	
	*K*_*eq*_ = 1.7 × 10^− 4^		
$HC0_{3\;}\overset{K^{f}}{\to }\;C0_{3}\;+H^{+}$	*K*_*eq*_ = 5.62 × 10^− 11^		28

## RESULTS AND DISCUSSION

### Inactivation of Pseudomonas putida using CO_2_ microbubbles with and without the ethanol addition

**Figure 2:**
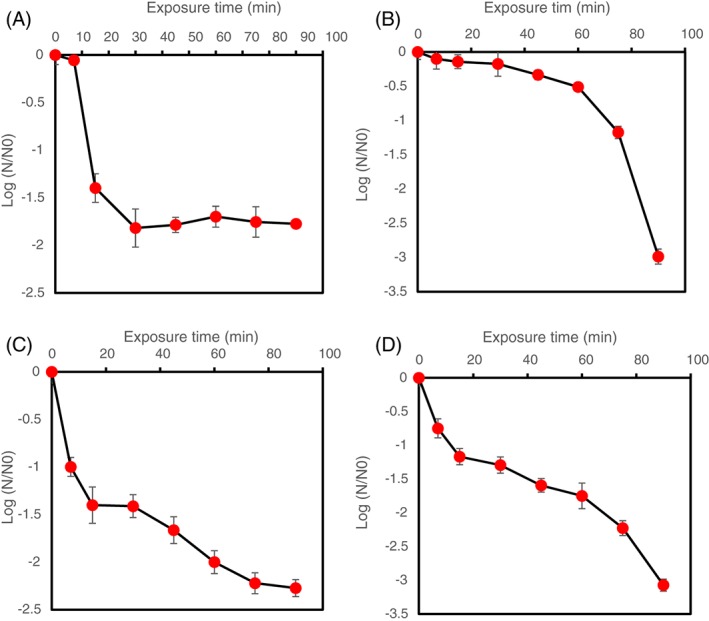
Survivor ratio of Pseudomonas putida after treatment with: (A) CO_2_ microbubbles; (B) CO_2_ microbubbles plus 2% (v/v) ethanol; (C) CO_2_ microbubbles plus 5% (v/v) ethanol; (D) CO_2_ microbubbles plus 10% (v/v) ethanol. Error bars depict standard deviation.

*Pseudomonas putida* cells were treated with CO_2_ microbubbles for 90 min at 100 mL min^−1^ flow rate and ∼1 bar. At equilibrium state, the inactivation efficiency was measured using two values, *D‐value* and *L‐value*. *D‐value* is defined as the time required for 1 log cycle reduction in microbial population, and calculated from the negative reciprocal of the slope of regression line from the straight part of the survivor curve 29 in Fig. 2(A). On the other hand, *L value* is the time during which the number of microbial cells remains constant before starting the inactivation of microorganisms.^30^ The *D‐value* for treatment with CO_2_ microbubbles was 64.8 min, while there was 2‐log reduction in *P. putida* population. In contrast, *L value* was 9.6 min. Two mechanisms are suggested to play a central role in this process: oxidative stress and the CO_2_ effects.

Regarding the oxidative stress, during the shrinkage and subsequent collapse of bubbles, some hydroxyl radicals are generated.^12^ In addition, the hydroxyl radicals generated are readily converted to the superoxide radicals and *vice versa*.^31^ Previously, *P. putida* was reported to go through an oxidative stress, when exposed to free radicals.^32^ Free radicals are reactive oxygen species (ROS), and are injurious species, reacting with different components of cellular systems such as lipids, proteins and DNA.^33^ Fundamentally, lipids seem to be the major targets for these species during an event of oxidative stress, and interestingly, the free radicals that are formed can directly react with the polyunsaturated fatty acids in the cell membrane, provoking lipid peroxidation. The latter reaction can change membrane properties and disrupt the membrane‐bound proteins.^34^ Amplification to this reaction occurs when more radicals are generated and more polyunsaturated fatty acids are broken down into other highly reactive products such as aldehydes. These highly reactive products can cause severe damage to vital compounds such as proteins.^35^

**Figure 3:**
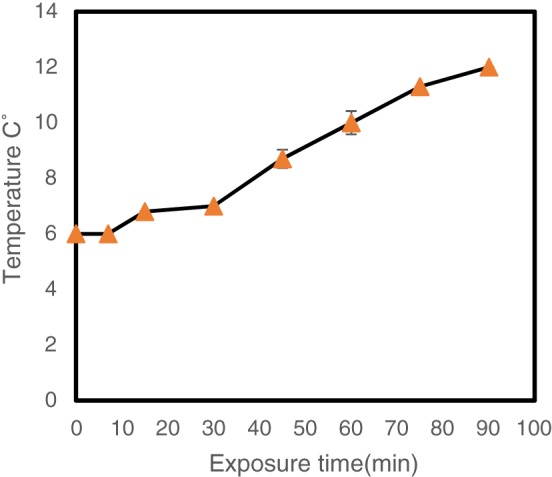
Temperature profile during CO_2_ enriched microbubbles inactivation process. Error bars depict standard deviation.

The mechanisms of the action of CO_2_ have been described in depth.^5^ CO_2_ is not a ‘natural product’ of the glucose metabolism pathway of *P. putida*, however, it can be produced during the catabolising of aromatic compounds by the *β‐Ketoadipate* pathway.^36^ All these factors work together to achieve the elevated log reduction observed in *P. putida* using CO_2_ microbubbles. In contrast, other microorganisms such as *Zymomonas mobilis* (another example of a gram‐negative bacteria) are expected to show a high degree of adaptation to CO_2_ as it is produced naturally during metabolism in both aerobic and anaerobic conditions.^37^, ^38^, ^39^ Figure 3 shows the temperature profile during the inactivation experiments.

Low temperature was used to increase the CO_2_ solubility in order to enhance the inactivation activity. At 0 °C, the CO_2_ solubility is around 1.3 mol L^−1^, decreasing to _∼_1 mol L^−1^ at 10 °C. Moreover, increasing the temperature to 20 °C will decrease the CO_2_ solubility to _∼_0.7 mol L^−1^.^40^ Figure 3 shows there was a gradual increase in temperature of the solution from 6 °C to 12 °C.

To enhance the inactivation activity of CO_2_ microbubbles, organic solvents, are employed. These solvents are toxic to some microbial cells due to their tendency to partition preferentially in the cytoplasmic membranes, increasing the fluidity of the cell membrane, and ultimately causing an increase in the nonspecific permeabilization of the cytoplasmic membrane.^41^, ^42^ Moreover, the fatty acid composition in the cytoplasmic membrane of microbial cells can be changed significantly by these membrane‐active solvents.^43^, ^44^ Isomerisation is a mechanism employed by *P. putida* to adapt its cytoplasmic membrane to ethanol toxicity. In this mechanism, the *Cis*‐unsaturated fatty acids are isomerised to Trans‐unsaturated fatty acids, and the bacterial cells become much more robust to the ethanol stress.^45^ Figure 2(B) shows the survivor ratio of *P. putida* cells after treatment with CO_2_ microbubbles combined with 2 % (v/v) ethanol for 90 min at 100 mL min^−1^ flow rate and _∼_1 bar.

Adding 2% ethanol enhanced the inactivation process, and caused ∼3‐log reduction in the microbial population after 90 min. Interestingly, the time during which the number of microbial cells remains constant‐‐*L valu*e‐‐ was_∼_30 min, _∼_4 times longer than the previous experimental set while the *D value* was almost identical in both experiments. Enhancement of the inactivation activity can be attributed to mechanisms of action of both ethanol and CO_2_ on the cytoplasmic membrane of *Pseudomonas* cells and the combined action of both elements. Both CO_2_ and ethanol work mainly on the fatty acid composition of the membrane.

Changing the composition of the cytoplasmic membrane is not straightforward and a certain threshold needs to be reached before observing any changes in the membrane. The exact duration to reach this threshold has not been studied yet for both CO_2_ and ethanol. However, previous studies on the changes in the fatty acids profile are helpful to predict the duration. For example, Mejia *et al*.,^46^ reported changes in the fatty acids profile of *Escherichia coli* (gram‐negative bacterium) after exposure to heat shock stress for 30 min. Another example is the study by Boylan *et al*.,^47^ where *Bacillus subtilis* (gram‐positive bacterium) was exposed to environmental stresses such as salt stress for 15–30 min to accumulate *β‐galactosidase*.

During the first 30 min, the level of the *Pseudomonas* population was almost constant before the number of bacterial cells gradually decreased with time until the CO_2_ concentrations reached the equilibrium state.

In addition, it has been suggested that increasing the amount of ethanol used (the organic solvent) could intensify the inactivation process with CO_2_ microbubbles. Therefore, the concentration of ethanol was increased to 5% (v/v) to test this hypothesis. Figure 2(C) shows the survivor ratio of *P. putida* after treatment with CO_2_ enriched microbubbles plus 5% ethanol. Interestingly, there was almost a 2.5 log reduction of *Pseudomonas* population after treatment. Further, it can be noticed that the time during which the cell number remained constant was much less than in the previous experiments, as well as the D‐value, which reached around 46.8 min. This observation might not be expected but on the other hand, the *L value* was much lower than in the previous experiment. While decreasing the log reduction in this treatment might have resulted from the increase in bacterial tolerance to ethanol, the microbial exposure to a sub‐lethal level of ethanol might promote a cellular response to encounter this stress. For example, Vanbogelen *et al*.,^48^ and Waston^49^ reported induced expression of stress proteins as a result of exposure to sub‐lethal levels of ethanol and the same proteins were expressed as a result of heat shock. Therefore, there might be a cross‐protective response induced to face environmental stresses in the same manner. Indeed, this cross‐protective response was seen previously in *Pseudomonas* spp, on exposure to different aromatic compounds and heat. In all three cases, stress shock proteins were produced.^50^

The concentration of additive (ethanol) was increased to 10% (v/v) to verify the combined effects of ethanol and CO_2_ and to enhance the whole inactivation process. It can be observed from Fig. 2(D) that there was around 3.5 log reduction in the bacterial population after this treatment. In addition, the D‐value for this experimental set was 82.8 min, while the L‐value was around 4.8 min. Both of these values were higher than the previous experimental set, and consistent with the original hypothesis. Indeed, the magnitude of ethanol toxicity is associated with its concentration used.^51^ Increasing the ethanol concentration to 10% can cause chemical stress to the bacterial cells, and this stress might be analogous to other stresses.^52^ As mentioned above, *P. putida* can evolve adaptative mechanisms as a response to stresses.^43^, ^48^ Therefore, it was speculated that increasing the ethanol concentration above a certain threshold could exceed the ability of *Pseudomonas* cells to tolerate and respond to the elevated level of toxicity, causing serious injuries to the cytoplasmic membrane and eventually failure to keep the biological system balanced. Another important concept to be considered is chaotropicity. Ethanol is known as a chaotropic solute, resulting in water stresses in bacteria at concentrations similar to levels in the environment.^53^ Hallsworth *et al*.^53^ showed that ethanol did not affect cell turgor, but instead, perturbed macromolecule–water interactions and thereby destabilized cellular macromolecules, and inhibited growth. This bacterium responded to ethanol chaotropicity by specifically up‐regulating the synthesis of proteins involved in stabilizing protein structure, in lipid metabolism, and in membrane composition.^53^ However, destabilization of macromolecules in the biological system is an elastic process in comparison with the specific inactivation (such as inactivation with carbon dioxide). Macromolecules destabilization can be reversed up to a critical thermodynamic point by using kosmotropicity solutes that increase entropy and affect hydration of macromolecules, such as trehalose.^53^ These solutes tend to order water, and strengthen electrostatic interactions within organic macromolecules.^54^, ^55^, ^56^

### CO_2_ concentrations during CO_2_‐enriched microbubbles sparging

**Figure 4:**
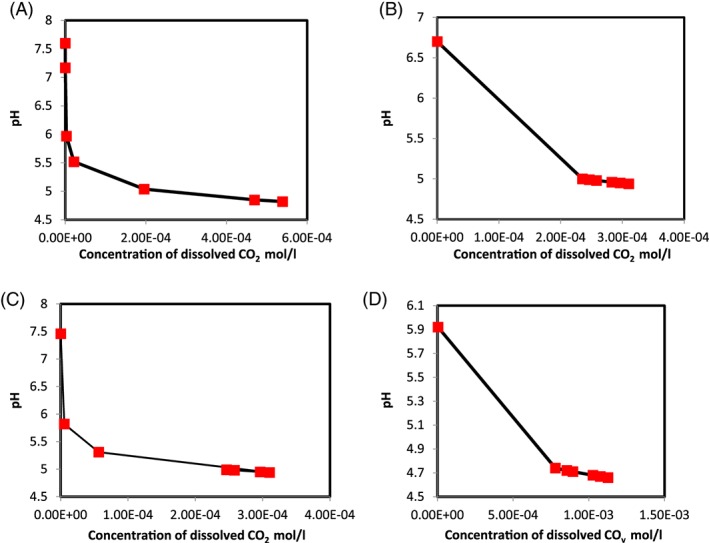
CO_2_ concentrations with different pHs observed during sparging. (A) CO_2_ microbubbles. (B) CO_2_ microbubbles plus 2% ethanol. (C) CO_2_ microbubbles plus 5% ethanol. (D) CO_2_ microbubbles plus 10% ethanol. Points are representative of triplicate results.

The CO_2_ concentration was estimated using Equation (10). Figure 4(A), (B), (C) and (D) present CO_2_ concentrations at different pHs for all experimental sets. From Fig. 4, it can be noticed that the inactivation process was increased in conjunction with increase in CO_2_ concentration. This observation can be explained by the fact that the elevated CO_2_ concentration tends to accumulate in the phospholipid bilayers of cytoplasmic membrane, causing an increase in the penetrated CO_2_.^21^, ^57^ Also, increasing the dissolved concentration of CO_2_ was accompanied with decreasing survivor ratio of *Pseudomonas* population as the inactivation efficiency depends on CO_2_ concentration.^58^

### Morphological changes on Pseudomonas putida cells using CO_2_ microbubbles with and without ethanol addition

**Figure 5:**
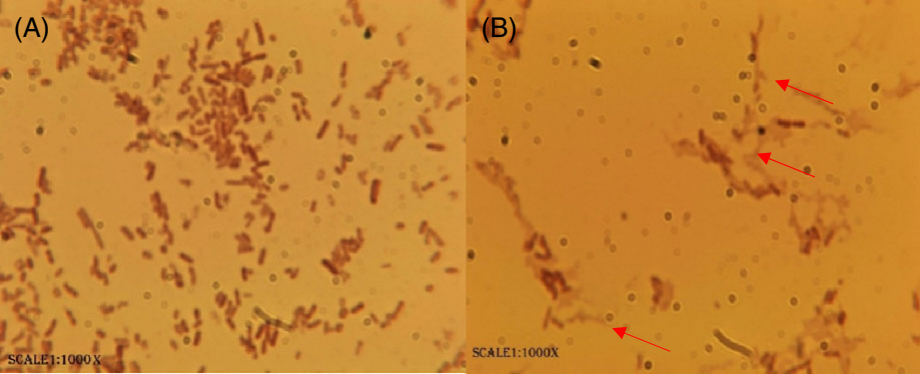
Numerical changes after treatment with CO_2_ microbubbles on 1000×. (A) Bacterial smears before sparging CO_2_. (B) Bacterial smear after treating with CO_2_‐enriched microbubbles for 90 min.

**Figure 6:**
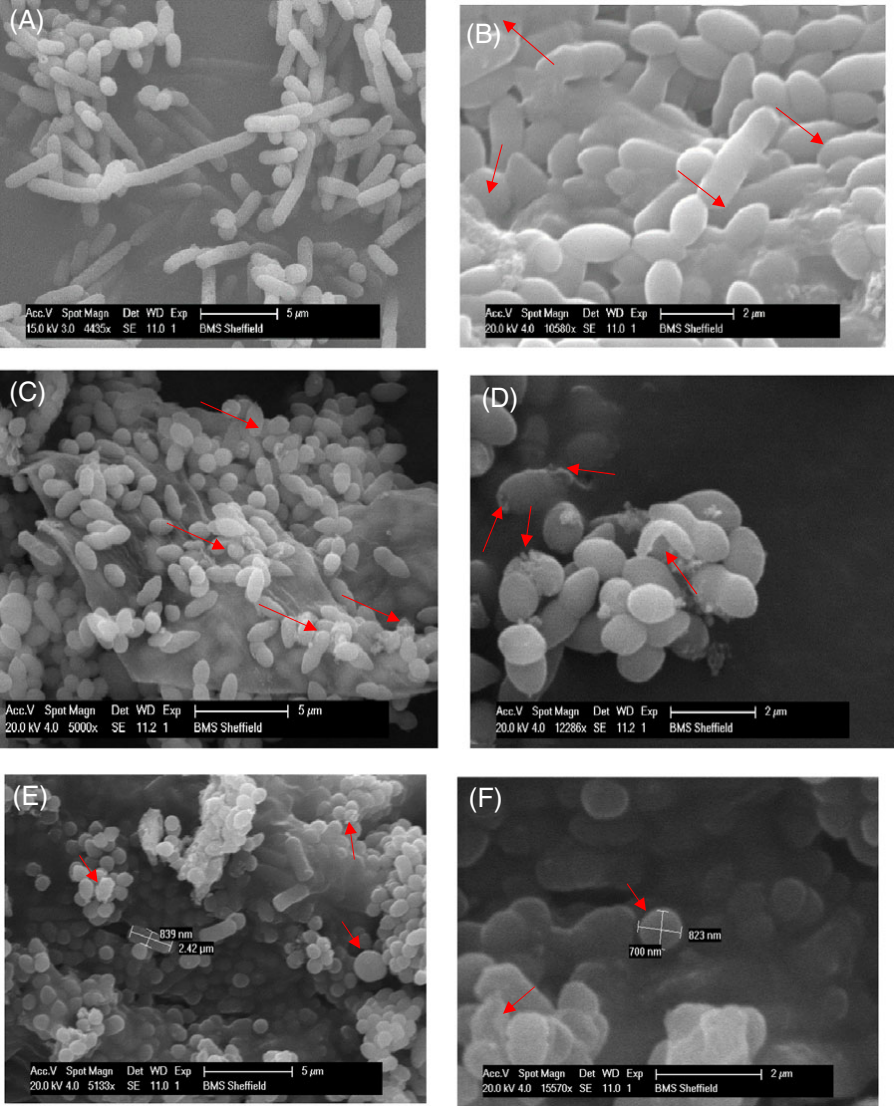
Morphological changes on (A) Pseudomonas cells before the inactivation process with CO_2_ microbubble plus 2% ethanol. (B) Pseudomonas cells after the inactivation process with CO_2_ microbubble plus 2% ethanol. (C) Pseudomonas cells before the inactivation process with CO_2_ microbubble plus 5% ethanol. (D) Pseudomonas cells after the inactivation process with CO_2_ microbubble plus 5% ethanol. (E) Pseudomonas cells before the inactivation process with CO_2_ microbubble plus 10% ethanol. (D) Pseudomonas cells after the inactivation process with CO_2_ microbubble plus 10% ethanol.

The inactivated cells with CO_2_‐enriched microbubbles treatment were examined using microscopy to observe the morphological/numerical changes in the bacterial cells after treatment (Fig. 5). There was a clear reduction in the number in comparison with the untreated cells. Further observation (Fig. 6) revealed changes in the morphology such as shortening and shrinkage of the cells.

Much research has been done on using supercritical CO_2_ for the inactivation of microorganisms. For example, it was hypothesized that application of supercritical conditions can facilitate CO_2_ penetration into the cell membrane, consequently expanding the microbial cells and causing cell disruption.^59^ Indeed, the concept behind using supercritical CO_2_ was originally described by Fraser,^60^ when bacterial cells were burst after injecting CO_2_ under high pressure. Thereafter, this concept was used to recover some cellular constituents such as intracellular enzymes and proteins.^61^ While many of the previous studies on inactivation with CO_2_ were achieved under high pressure and elevated temperature (_∼_73 bars and 31.1 °C) to reach the supercritical state,^62^ the current study was achieved under relatively low‐pressure CO_2_ (_∼_1 bar) and low temperatures (6–12 °C). This suggests that high pressure and temperature are not the only factors affecting cell lysis by CO_2_ application.

Figure 6 shows morphological changes to the *Pseudomonas* cells after treatment with CO_2_ microbubbles plus ethanol as an additive. In Fig. 6(B), it is obvious that the *Pseudomonas* cells were aggregated after the treatment with CO_2_‐enriched microbubbles plus 2% ethanol. Many conditions can provoke microbial flocculation such as substrate acquisition, slow growth or starvation, physical and chemical stress and aggregation to protect against predation.^63^ Moreover, formation of microbial flocs can also help increase the metabolic activity of the stressed cells^64^ and enhance the microbial resistance to toxic compounds such as biocidal compounds.^65^, ^66^, ^67^ The changes in cell shapes can also be noticed in Fig. 6(B) in comparison with the normal bacterial cells in Fig 6(A) (the control), when *Pseudomonas* cells were observed to transition from rod cells to coccus cells. This transition might increase attachment of the bacterial cells, consequently leading to flocculation.^68^ Occasionally, the size reduction is accompanied by an increase in population under various environmental stresses.^68^, ^69^, ^70^, ^71^, ^72^, ^73^ Interestingly, the decrease in cell size was not accompanied with an increase in the bacterial population in the current study, as shown in Fig. 2(B). Changing the cells shape from rod to round shape increases the cell surface area to volume ratio; a useful metabolic response under starvation stress condition. Bacterial cells are known to adjust in order to effectively transport nutrients with minimum energy consumption.^74^ However, alternating the *Pseudomonas* cells shape from rod to round shape in the current study is likely a consequence of changes due to the environmental stresses mentioned above.

Figure 6(D) shows the morphological changes that occurred in the *Pseudomonas* cells as a result of the treatment with CO_2_ enriched microbubbles plus 5% ethanol. It is apparent that the cells have lesions with loss of membrane integrity. Cell injury and death were previously observed, due to high pressure CO_2_ application.^75^ The current study, however, was conducted under low pressure (∼1 bar), and observing the same morphological changes on the bacterial cells is suggestive of an inactivation and subsequent cell lysis capability of CO_2_ according to the mechanisms reviewed previously.^5^ Using CO_2_ under high pressure can enhance the solubility of CO_2_ and consequently, accelerate cell injury and death.^75^ These changes are irreversible.^4^ Therefore, application of high pressure is a way to enhance the CO_2_ inactivation activity but by no means the only factor responsible for inactivation.

The morphological changes during treatment with the CO_2_‐enriched microbubbles plus 10% ethanol are presented in Fig. 6(E) and (F). Increasing the ethanol concentration can increase the membrane permeability of the bacterial cells, which is associated with chemical activities, inducing narcosis in biological systems.^21^ This effect is followed by leakage of protons and some vital ions such as potassium ions from the cytoplasmic membrane of the bacterial cells.^76^, ^77^ Interaction of these compounds with the phospholipid bilayers of the cytoplasmic membrane could cause substantial changes in the membrane structure. For example, lipophilic compounds tend to accumulate in the hydrophobic part of the cytoplasmic membrane, disturbing the interaction between acyl chains of the phospholipid bilayers as well as changing the fluidity of the membrane. Eventually swelling of the phospholipid bilayers of the bacterial cells can occur, resulting in a ball‐like shape.^78^

## CONCLUSION

*Pseudomonas Puitda* KT2240 was used as a model for gram‐negative bacteria in the current study. Survivor ratio after each experiment was calculated after streaking the bacterial samples on nutrient agar plates. The initial log reduction with CO_2_ microbubbles alone was around 2 Log, while best Log reductions were achieved with 10%, 2% and 5% ethanol, respectively. Using microbubble technology for CO_2_ sparging caused both an oxidative stress and disturbance to the biological system of *P. putida* cells. Moreover, addition of ethanol amplified the activities of CO_2_ microbubbles and decreased the survivor ratio of *P. putida*. Several morphological changes were observed after each treatment, and these changes ranged from changing the cell shape from rod to round, lesions appearing on the bacterial cells, severe injurious signs and cell death.
